# Plant-soil feedbacks promote coexistence and resilience in multi-species communities

**DOI:** 10.1371/journal.pone.0211572

**Published:** 2019-02-11

**Authors:** Keenan M. L. Mack, Maarten B. Eppinga, James D. Bever

**Affiliations:** 1 Department of Biology, Indiana University, Bloomington, Indiana, United States of America; 2 Department of Environmental Sciences, Copernicus Institute of Sustainable Development, Utrecht University, Utrecht, the Netherlands; 3 Department of Geography, University of Zurich, Zurich, Switzerland; 4 Department of Ecology and Evolutionary Biology and Kansas Biological Survey, University of Kansas, Lawrence, Kansas, United States of America; National Taiwan University, TAIWAN

## Abstract

Both ecological theory and empirical evidence suggest that negative frequency dependent feedbacks structure plant communities, but integration of these findings has been limited. Here we develop a generic model of frequency dependent feedback to analyze coexistence and invasibility in random theoretical and real communities for which frequency dependence through plant-soil feedbacks (PSFs) was determined empirically. We investigated community stability and invasibility by means of mechanistic analysis of invasion conditions and numerical simulations. We found that communities fall along a spectrum of coexistence types ranging from strict pair-wise negative feedback to strict intransitive networks. Intermediate community structures characterized by partial intransitivity may feature “keystone competitors” which disproportionately influence community stability. Real communities were characterized by stronger negative feedback and higher robustness to species loss than randomly assembled communities. Partial intransitivity became increasingly likely in more diverse communities. The results presented here theoretically explain why more diverse communities are characterized by stronger negative frequency dependent feedbacks, a pattern previously encountered in observational studies. Natural communities are more likely to be maintained by strict negative plant-soil feedback than expected by chance, but our results also show that community stability often depends on partial intransitivity. These results suggest that plant-soil feedbacks can facilitate coexistence in multi-species communities, but that these feedbacks may also initiate cascading effects on community diversity following from single-species loss.

## Introduction

Understanding the maintenance of biodiversity through the coexistence of apparent competitors is one of the central challenges in ecology. Ecological theory suggests that negative frequency dependent feedbacks preventing exclusion of the least fit species is a necessary requirement for coexistence [1,2]. In plant communities, such feedbacks were traditionally thought to be the result of competition for abiotic resources [3–5]. However, recent evidence suggests that biotic interactions, particularly interactions with soil micro-organisms, can generate frequency dependent negative feedback that plays an important role in plant community structure [6,7]. As a plant grows, its presence at a particular site promotes compositional shifts in the microbial community under and around it and the composition of that microbial community in turn feeds back on the growth and reproduction of that plant, its neighbors and/or the next plant to grow at that site. This particular type of frequency dependent feedback is referred to as plant-soil feedback (PSF). The analyses of PSFs have proven a useful framework for investigating how soil organisms affect plant community dynamics.

PSFs can be negative or positive. Negative PSF occurs when the presence of a particular species modifies the soil community in a way that reduces the competitive ability of other individuals of the same species at or near the site relative to heterospecifics. In contrast, positive PSF occurs when modification of the soil community increases conspecific competitive ability relative to heterospecifics. Empirical work indicates that feedbacks on plant growth are commonly negative [8–10] which is consistent with PSFs contributing to plant species coexistence [11–13]. Spatial signatures of negative PSFs have also been documented in forested systems as the performance of seedlings decline near adult conspecifics [14]. Moreover, negative PSFs were found to correlate with relative abundance in plant communities [9,14,15] and theoretical work suggests that this correlation occurs when negative feedbacks structure plant communities [15–17]. Together this body of work suggests that soil community dynamics can contribute to plant species coexistence.

Theoretical studies have shown that negative PSF can facilitate the coexistence of strong competitors [12, 18], and empirical observations suggest that more diverse communities are characterized by more strongly negative PSFs [19]. Little is known, however, about the relation between the strength of PSFs and community robustness to species loss. Classic theory suggests that as a community’s species richness increases, its vulnerability to cascading diversity loss due to species removal may also increase [20–22]. Recent work on networks of species interactions suggest that persistence and robustness to species loss are enhanced by increased diversity in mutualistic networks, but the opposite is true in antagonistic ones [23]. How these insights relate to communities structured by negative PSFs has not yet been investigated.

Previous modeling efforts on frequency dependent feedbacks have primarily focused on the analysis of coexistence of pairs of species [11,12,18], simulations of multiple species coexistence [14, 16, 18,24], or analysis of invasion of communities dominated by a single plant species [12,25,26]. Despite these efforts, there has been limited attention for distinguishing qualitatively different network structures that can be generated by frequency dependent feedbacks, and how the specific network structure affects coexistence, invasibility, and resilience to extinction [27]. This limited exploration of the dynamics of multispecies plant communities structured by negative feedback is particularly problematic given the evidence of the role negative feedbacks play in the maintenance of diversity and the success of invasions into these diverse communities.

In this study, we develop and graphically analyze a generic two and three species model, to find the conditions under which negative frequency dependent feedbacks facilitate coexistence within communities. Previous 2 species feedback models have identified the conditions for coexistence narrowly such that the growth of a species in the presence of itself is always less than that of its competitor, which we will refer to as strict pair-wise negative feedback. However, by analyzing the 3-species model, we identify and describe how intransitivity relaxes this condition for coexistence, revealing novel routes to coexistence created by keystone competitors. The 3-species model identifies the potential role in coexistence of intransitivity, the condition when a species grows better in the presence of itself than at least one other competitor, but coexists with that competitor because of the presence of a third species. We then apply the framework to real plant communities by parameterizing the model with the results from PSF studies. The benefit of using PSF studies is that it establishes a clear mechanism for frequency dependent feedback and a method for quantifying feedbacks empirically. Using numerical simulations of communities with 3, 4 and 5 species, we look at how species richness in communities structured by negative feedbacks affects stability. We describe the feedback conditions under which an introduced species will successfully establish in a native community, and whether the invader will reduce native diversity or coexist with the native species and become naturalized. We also investigate how communities with different feedback structures ranging from strict pair-wise negative feedbacks to strict intransitivity differ in their robustness to extinction.

### Methods and model description

The starting point of our framework is the plant-soil feedback model developed by Bever et al. (1997). This model describes how each plant species amplifies a subset of soil community organisms, which in turn affects growth of the host plant species and its competitors. The conspecific and heterospecific soil community effects are represented by the growth parameter *σ*, which represents the (per capita) soil community effect on the plant’s relative growth rate. Pair-wise soil community effects are indexed so that *σ*_*ij*_ represents the soil community effect induced by plant species *j* and its effect on the growth of plant species *i* [28]. We consider, *σ* ≥ 0, meaning that the most detrimental effect a species' soil community on the growth and colonization potential of another species is no net growth. These soil community effects *σ* capture processes that are not included in standard Lotka-Volterra competition models, and previous studies have focused on how Lotka-Volterra dynamics may be altered when soil community effects are included [12,18,28]. In this study, we focus on the dynamics that can be generated exclusively through soil community effects. Under this constraint, the realized growth rate of plant species *i* in a two species model is defined as:

$$w_{i}=\sigma_{ii}S_{i}+\sigma_{ij}S_{j},$$ (1)

where *S*_*i*_ and *S*_*j*_ represent the proportion of soil community effects attributable to plant species *i* and *j* respectively. Bever et al. (1997) derived a relationship between the change in frequency of plant species *i*, *P*_*i*_, and the realized growth rate of its competitor species *j*:

$$\frac{dP_{i}}{dt}=P_{i}P_{j}\left((\sigma_{ii}-\sigma_{ji})S_{i}–(\sigma_{jj}-\sigma_{ij})S_{j}\right)$$ (2)

Although it is challenging to directly measure the rates at which plant species create different soil communities[29], it is reasonable to assume that these dynamics occur relatively rapidly [30]. Moreover, the carrying capacity of a soil community associated with a specific plant host (i.e. a ‘specific soil community’ c.f. Eppinga et al. 2006) can be assumed to be proportional to the current density of that host. These assumptions motivate a quasi-steady state approach to describing specific soil community density dynamics:

$$\frac{dR_{i}}{dt}=R_{i}\left(1-\frac{N_{i,max}}{N_{i}}\frac{R_{i}}{R_{i,max}}\right)=0$$ (3)

In which *N*_*i*_ is the density of plant species *i* and *R*_*i*_ is the density of its associated specific soil community. Note that plant frequencies and densities are related as $P_{i}=\frac{N_{i}}{\sum_{j=1}^{n}N_{j}}$. Specific soil community densities and the proportion of soil community effects attributed to this community are related as:

$$S_{i}=\frac{{\mu_{i}R}_{i}}{\sum_{j=1}^{n}\mu_{j}R_{j}}$$ (4)

with *μ*_*i*_ as the per capita effect of specific soil community *i* on plant growth. Further, *N*_*i*,*max*_ is the carrying capacity of species *i* and *R*_*i*,*max*_ is the carrying capacity of its associated specific soil community. Solving Eq (3) then yields the density to which the specific soil community will develop [30]:

$${\hat{R}}_{i}=\frac{R_{i,max}}{N_{i,max}}N_{i}$$ (5)

In this study, we assume that specific soil community densities are proportional to the density of their host, meaning that we set $\frac{\mu_{i}R_{i,max}}{N_{i,max}}=\frac{{\mu R}_{max}}{N_{max}}$, i.e. this ratio is set equal for all species. Combining Eqs (4) and (5) under these assumptions, we can then write:

$$S_{i}=\frac{\mu_{i}\frac{R_{i,max}}{N_{i,max}}N_{i}}{\sum_{j=1}^{n}\mu_{j}\frac{R_{j,max}}{N_{j,max}}N_{j}}=\frac{\mu \frac{R_{max}}{N_{max}}}{\mu \frac{R_{max}}{N_{max}}}\frac{N_{i}}{\sum_{j=1}^{n}N_{j}}=P_{i}$$ (6)

Through this approach we focus on the net effects that soil communities exert on plant growth (through the model parameters *σ*_*ij*_), rather than the explicit characterization of the differences in magnitude of specific soil communities, which are difficult to quantify empirically [11, 29, 31]. In contrast, the parameters *σ*_*ij*_ can be quantified directly using either pot experiments [11] or field estimates [19, 32]. As the parameters *σ*_*ij*_ control relative fitness, the parameterization can be done in multiple ways. One way is to use *σ* as modifiers of realized growth rates, with values <1 indicating negative effects, values >1 indicating positive effects and a value of 1 indicating a neutral soil community effect [19]. Using this assumption, we obtain a generic, phenomenological description of frequency dependence, in which a plant species’ realized growth rate is defined as:

$$w_{i}=\sigma_{ii}P_{i}+\sigma_{ij}P_{j}$$ (7)

Substitution of Eq 5 in Eq 2 then yields:

$$\frac{dP_{i}}{dt}=P_{i}P_{j}(w_{i}-w_{j})$$ (8)

Species frequencies range between 0 and 1, with *P*_*i*_+*P*_*j*_ = 1. Hence, we can rewrite Eq 6 as:

$$\frac{dP_{i}}{dt}=P_{i}P_{j}(w_{i}-w_{j})=P_{i}(w_{i}P_{j}–w_{j}P_{j})=P_{i}(w_{i}-w_{i}P_{i}–w_{j}P_{j}).$$ (9)

This two species model is easily expandable to a general form that describes a system with any number of plant species, *n*, as:

$$\frac{dP_{i}}{dt}=P_{i}(w_{i}-\sum_{j=1}^{n}(w_{j}P_{j}))$$ (10)

where

$$w_{i}=\sum_{j=1}^{n}\sigma_{ij}P_{j}.$$ (11)

Eqs (8) and (9) describe the so-called replicator equation, which originated from evolutionary game theory, and has been applied in other fields such as economics [33–35]. Through the application of Cramer’s rule, the internal coexistence equilibrium for communities of *n* species can be described as:

$${\hat{P}}_{i}=\frac{det(M_{i})}{\sum_{j=1}^{n}det(M_{j})}$$ (12)

In which ***M*** indicates the interaction matrix ***M*,** in which *σ*_*jk*_ is entry on the *j*^th^ row and *k*^th^ column. More specifically, the matrix ***M***_***i***_ indicates the matrix **M**, in which the *i*^th^ column has been replaced by a column vector of ones (and of length *n*). Eq (10) can be used to assess the feasibility of the coexistence equilibrium (as this requires 0 < ${\hat{P}}_{i}$ < 1 for all species i, see Eppinga et al. 2018 for more details). The stability of this internal coexistence equilibrium can then be assessed by means of standard eigenvalue analysis (here we used the eigenvalue function ‘eig()’ as implemented in Matlab (version 9.0, Mathworks 2016)). However, numerical simulation (see Appendix A) revealed that eigenvalue analysis is not a sufficient means to evaluate persistence of all species within the communities. Therefore, while the magnitude of the dominant eigenvalue can indicate the degree of stability and resilience of the coexistence equilibrium (e.g. [36]), in the analyses described below species persistence will also be examined via numerical integration (see details below).

#### Simulation of randomly assembled communities

We applied the model framework to communities with richness starting between two and five species. We randomly assembled communities by choosing *σ*_*ij*_*-*values from a uniform distribution between 0 and 1 for each species, using the random number generator as implemented in Matlab (version 9.0, Mathworks 2016). Using the general form of the model (Eqs 8 and 9), species started with nearly equal frequencies, and the communities were allowed to change for 10,000 time units. Because population oscillations (see Appendix A) were possible, the frequency of each species was averaged over the last 100 time units and recorded. When the average frequencies of all species in the community were greater than 0.0001 during this interval, coexistence was assumed. 5,000 communities were randomly assembled at each species richness level and the proportion that demonstrated coexistence was recorded by numerically simulating the population dynamics as well as solving the equilibrium densities analytically (Eq 12), and determining the dominant eigenvalue of this equilibrium by evaluating the community matrix at this point. Coexistence type was determined for each community by scoring the proportion of species whose σ-value for its own environment was less than other species grown in that environment. If all the species matched that criterion, the community structure was classified as strict pair-wise negative feedback, and if none of the species did worst in an environment dominated by itself, the community was classified as strictly intransitive. Following Bever et al. (1997), the average pair-wise feedback for each species across each simulated community was calculated according the formula:

Average feedback for species *i* $=\sigma_{ii}+\sum_{j\neq i}^{n}\left(\sigma_{jj}-\sigma_{ij}-\sigma_{ji})/(n-1\right)$ Although the average pair-wise feedback does not provide a formal condition for coexistence in multi-species communities, it is correlated to community structure, and to community stability as well [19].

Stably coexisting communities assembled at random were challenged by an invader with randomly assigned *σ*_*ij*_ values between 0 and 1 (using the same method as described above) in order to test the effect of community richness on a community’s ability to resist invasion. For each species level (i.e. 2, 3, 4, or 5 species), we generated 30 replicates for each coexistence type, that is the number of species within the community with strict pairwise negative feedback. Each of these replicates were challenged sequentially by 1,000 invaders. The proportion of successful invasions and mean proportion of richness retained following a successful invasion after 10,000 time units were recorded. These same replicates were also tested for resilience by removing each species from the community and recording the subsequent loss of diversity after 10,000 time units. All simulations were carried out in Matlab (version 9.0, Mathworks 2016), using forward Euler integration with a timestep of 0.001. All statistical analyses were performed in SAS. A GLM with species level and community coexistence type as predictors was used to test significance and reported means are LS means unless otherwise noted.

#### Resampling and simulation of experimental communities

The 5000 randomly assembled communities at each richness level were compared to 2, 3, 4 and 5 species subsamples of experimental communities described plant-soil feedback studies from the literature. Unlike previous meta-analyses on this type of frequency dependent feedbacks [10], we used only studies with three or more plant species arranged in a full-factorial design. Eighteen published studies met this criterion, and subsampling within each of these experimental communities resulted in 177 two species combinations, 231 three species combinations, 211 four species combinations, and 137 five species combinations [8, 14, 29, 37–51]. The study systems ranged from old field, to tallgrass prairie, to tropical forest and all had unique sites in North America, Central America and Europe. The data were relativized within each study to remove effects due to species differences in size, and thereby, isolate growth rate differences. This was done by rescaling the mean size of each species so that every species had the same mean size and using only the deviations from this mean as the σ-values:

$${\tilde{\sigma }}_{ij}=\sigma_{ij}-\frac{1}{n}\sum_{k=1}^{n}\sigma_{ki}+\frac{1}{n^{2}}\sum_{l=1}^{n}\sum_{k=1}^{n}\sigma_{kl}$$ (13)

In which ${\tilde{\sigma }}_{ij}$ indicates the relativized value. In Eq (13), the second term on the right-hand side depicts the average effect that a species exerts on all species in the community. Hence, the first two terms jointly quantify the deviation of this mean species-averaged effect. The third term on the right-hand side indicates the average effect that all species exert on all species in the community. As the deviations sum up to zero, this approach than ensures that the average effect exerted by each species is the same, effectively removing size-based differences. Through this procedure, any effects due to differences in the average plant size were eliminated. By resampling combinations of species from the published feedback studies and simulating their dynamics in isolation, we were able to generate observed measures of coexistence for a range of community sizes to be compared with the expected coexistence generated by random assembly. The simulation procedure for the empirical communities, including the invasions and species removal experiments, was identical to the procedure used for randomly assembled communities (as described above). Hence, this study design enables a systematic comparison of the stability, resilience and robustness to invasion of experimental communities, as compared to randomly assembled communities.

## Results

### Analysis of two species model

We begin our more detailed analysis by walking through the 2-species solution which recovers previous results [11, 29] because we will be using the same graphical analysis for the 3-species case. This graphical analysis is a useful tool as it provides an intuitive framework for thinking about how partial intransitivity can stabilize multi-species communities. Invasion criteria for the 2-species case can be found by assuming a near monoculture of one species and looking at the growth rate of the other species introduced under that condition. When *P*_*1*_ = 1, species 2 can invade when (1/*P*_*2*_)*dP*_*2*_*/dt*> 0, or when *w*_*2*_>*w*_*1*_. This result holds more generally: a species *i* with $w_{i}>\hat{w}$ (as defined in Eq 9) will be able to invade the community of species coexisting with $\hat{w}$. Simplifying Eq 1 given that *P*_*1*_ = 1 and *P*_*2*_ = 0, and substitution into the inequality *w*_*2*_>*w*_*1*_, we find that species 2 will invade species 1 when *σ*_21_ > *σ*_11_. When two species can reciprocally invade each other, they will coexist. For instance, species 1 and 2 coexist when both *σ*_12_ > *σ*_22_ and *σ*_21_ > *σ*_11_. Biologically, this inequality indicates that coexistence occurs when each species' effect on themselves is more negative than their effect on competitors.

**Fig 1:**
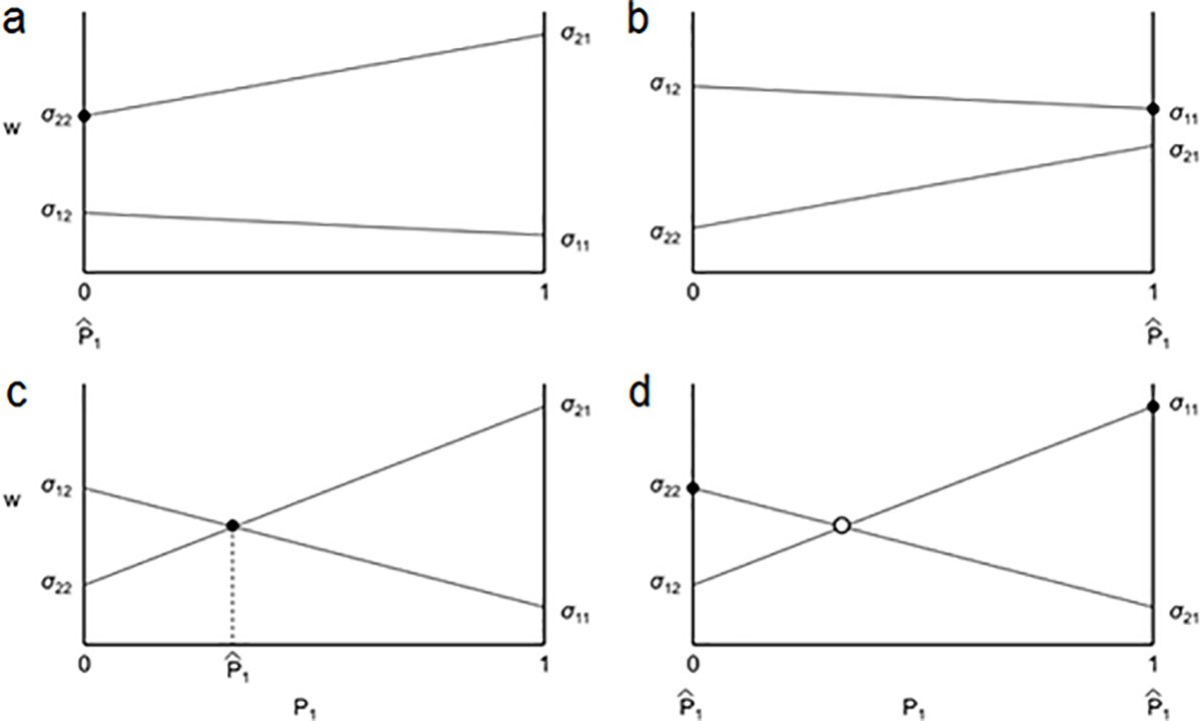
Plot of fitness (*w*) against the proportion of species 1 (*P*_*1*_). a) The fitness of species 2 is always greater than species 1 and therefore excludes species 1. b) The fitness of species 1 is always greater than species 2 and therefore excludes species 2. c) Species 2 has lower fitness than species 1 in a community dominated by species 2, however, species 1 has lower fitness than species 2 in a community dominated by species 1. This illustrates coexistence maintained by direct negative feedback. d) Each species has higher fitness in a community dominated by itself. Which species ultimately dominates depends on starting densities.

**Fig 2:**
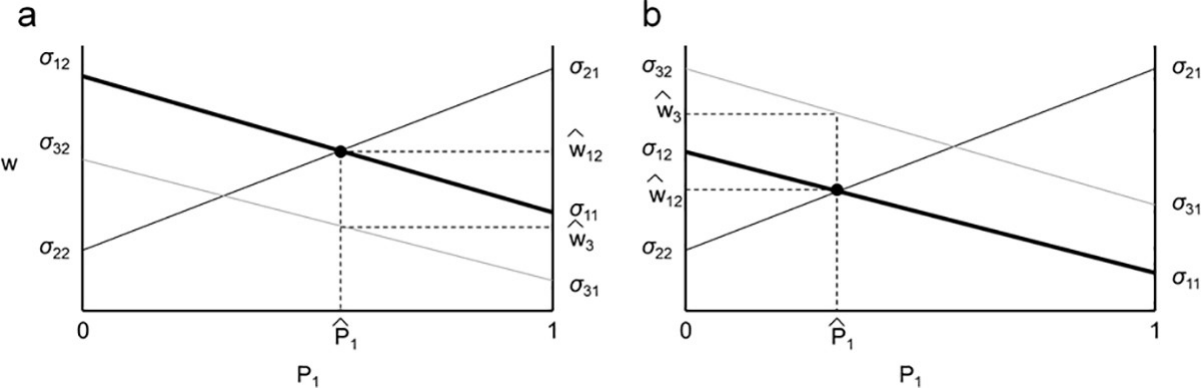
Plots showing the invasion conditions for a third species into a two-species community. a) The growth rate of species 3 (*w*_*3*_) is less than *w*_*1*_ and *w*_*2*_, therefore species 3 will be excluded. b) *w*_*3*_ is greater than the equilibrium growth rates of species 1 and 2, therefore species 3 invades the community.

By plotting the fitness functions, and finding the *σ*_*ij*_-values at the intercepts in the monoculture conditions, the equilibrium two-species dynamics can easily be visualized (Fig 1). When both *σ*_11_ > *σ*_21_ and *σ*_12_ > *σ*_22_, species 1 has a greater growth rate than species 2 when the proportion of sites occupied by species 1 (*P*_*1*_) is close to zero (i.e. *P*_*2*_ is close to 1), but also has a greater growth rate when *P*_*1*_ is close to 1 (Fig 2). In these cases, species 1 excludes species 2. When both *σ*_12_ > *σ*_22_ and *σ*_21_ > *σ*_11_, species 1 *i* has a greater growth rate than species 2 when *P*_*1*_ is close to zero, however now species 2 has a greater growth rate when *P*_*1*_ is close to 1 (Fig 1). Thus, each species can invade the other when rare, leading to stable coexistence and the intersection of the two lines represents the equilibrium frequency of species 1 ($\hat{P_{1}}$). This is the two species case of what we will refer to as strict pair-wise negative feedback. When both *σ*_11_ > *σ*_21_ and *σ*_22_ > *σ*_12_, species 2 has a greater growth rate than species 1 when *P*_*i*_ is close to zero, but species 1 has a greater growth rate when *P*_*1*_ is close to 1 (Fig 1). In this case, neither species can invade the other and initial frequencies of species 1 and 2 will determine which species will exclude the other.

### Analysis of three species model

We can analyze conditions for invasion of a 2-species community by a third species by analyzing each edge of a triplot independently (i.e. where the third species has a frequency near zero). Like the two species case presented above, when each species can invade the other two species, three-species coexistence is possible. If all 2-species equilibria are stable, these invasion conditions are also sufficient for coexistence (see Eppinga et al. 2018 for more details). Therefore, in order to find invasion criteria for the whole system, we need to establish invasion criteria for each of the two-species communities. Notice that when species 1 and species 2 coexist, w_1_ = w_2_ at $\hat{P_{1}}$ which we call $\hat{w_{12}}$. Using Eq 3,

$$\frac{dP_{3}}{dt}=P_{3}(w_{3}-w_{1}P_{1}-w_{2}P_{2}-w_{3}P_{3}).$$ (14)

Simplifying, remembering that P_3_ ≈0, P_1_ = $\hat{P_{1}}$, P_2_ = $\hat{P_{2}},\hat{P_{2}}$ ≈ (1 – $\hat{P_{1}}$), and w_1_ = w_2_ at $\hat{w_{12}}$, we get,

$$\frac{1}{P_{3}}\frac{dP_{3}}{dt}=w_{3}-\hat{w_{12}}.$$ (15)

This means that species 3 will not be able to invade a stable community of species 1 and 2 when $w_{3}<\hat{w_{12}}$ (as shown in Fig 2A), but will be able to invade when $w_{3}>\hat{w_{12}}$ (Fig 2B). Again, remembering that P_3_ ≈0, P_1_ = $\hat{P_{1}}$, P_2_ = $\hat{P_{2}},\;\hat{P_{2}}$ ≈ (1 – $\hat{P_{1}}$), using Eq 9 for w_1_ at the two-species equilibrium, and subsequent rearranging yields:

$$\frac{\sigma_{12}-\sigma_{32}}{\sigma_{22}-\sigma_{12}}<\frac{I_{13/12}}{I_{12}}.$$ (16)

Where *I*_12_ = σ_11_−σ_12_−σ_21_+σ_22_ and represents a measure of net pair-wise feedback between species 1 and 2, and *I*_13/12_ = σ_12_−σ_11_−σ_32_+σ_31_ and represents a measure of net dynamics between species 1 and 3 given that the environment is dominated by species 1 and 2. Alternatively, given that w_1_ = w_2_ at $\hat{w_{12}}$, the inequality can also be solved for w_2_ at the two-species equilibrium, which yields:

$$\frac{\sigma_{21}-\sigma_{31}}{\sigma_{11}-\sigma_{21}}<\frac{I_{23/12}}{I_{12}}.$$ (17)

Where *I*_23/12_ = σ_22_−σ_21_−σ_32_+σ_31_ and represents a measure of net dynamics between species 2 and 3 given that the environment is dominated by species 1 and 2. This confirms the importance of the pair-wise interaction terms in determining coexistence previously shown [11]. This also shows however, that in multispecies communities, there is a third-party interaction term that is important for determining coexistence. Notice that fulfilling inequalities (16) and (17) does not require strict pair-wise negative feedback, i.e. *σ*_*ii*_ < *σ*_*ij*_ and *σ*_*ii*_ < *σ*_*jk*_. Hence strict pair-wise negative feedback is not a necessary requirement for coexistence in the 3-species system.

**Fig 3:**
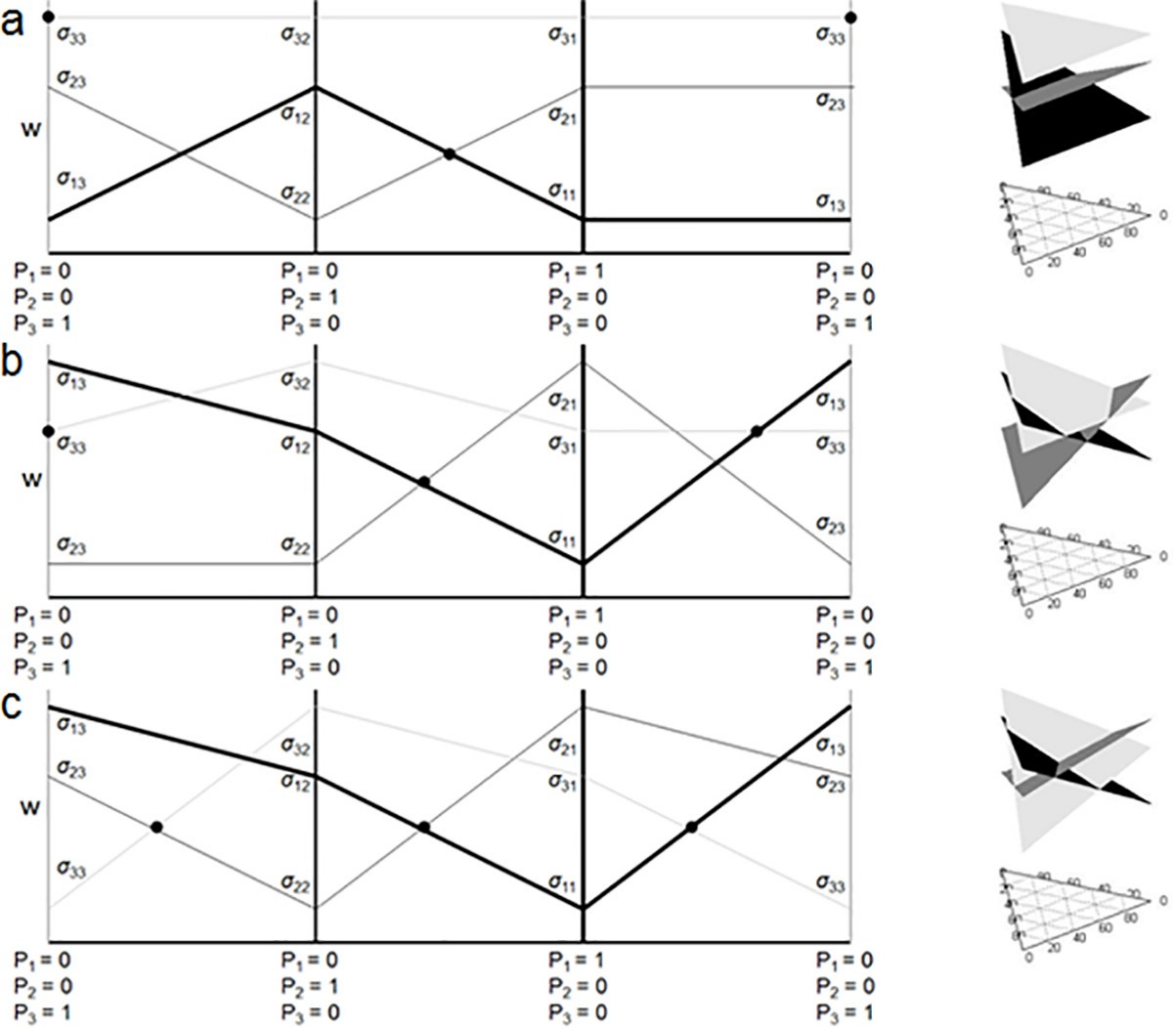
3D triplot of the surface representing the fitness of each species (species 1 = light grey, species 2 = dark grey, species 3 = black) given the relative proportions of each species. a) Species 3 can invade and exclude both species 1 and 2, b) Species 3 excludes species 2 and coexists with species 1, and c) Species 3 is naturalized and coexists with species 1 and 2. Panels a, b, and c are 2D representations of the corresponding 3D plots showing the edge conditions.

Following the graphical method presented above, we can expand the analysis to three species. Solving for each species’ *w*-value at equilibrium in each of the single species conditions gives three intercepts that allow us to plot a plane for each species on a triplot with *w* plotted on the *z*-axis (Fig 3). Coexistence is only possible when the 3 planes intersect where *P*_*1*_, *P*_*2*_ and *P*_*3*_> 0. If species 3 can invade a stable equilibrium with species 1 and 2 present, i.e. *w*_*3*_> $\hat{w_{12}}$, three qualitatively different outcomes are possible. First, species 3 can exclude both species 1 and 2 (Fig 3A). When *σ*_31_ > *σ*_21_ and *σ*_31_ > *σ*_11_, as well as *σ*_32_ > *σ*_22_ and *σ*_32_ > *σ*_12_, the growth rate of species 3 at equilibrium coexistence for species 1 and 2 will inevitably be higher than the other two species, therefore species 3 will invade. If *σ*_33_ is also greater than both *σ*_23_ and *σ*_13_, the other two species will be unable to reciprocally invade (Fig 3A).

Second, species 3 can coexist with either species 1 or 2 while excluding the other (Fig 3B). Like the previous case, if the growth rate for species 3 is higher than the equilibrium growth rate at coexistence of species 1 and 2, then species 3 will invade. However, unlike the previous case, if *σ*_33_ < *σ*_23_ or *σ*_33_ < *σ*_13_, then one of the other 2 species will be able to invade species 3 as it approaches fixation, therefore allowing coexistence with that other species. If at that two-species equilibrium the growth rate of the species not at equilibrium is less than the equilibrium growth rate of species 3 and the other species, then that third species will be excluded and only species 3 and the other species will remain (Fig 3B).

Or third, all three species can coexist (Fig 3C). Again, like before, if the growth rate of species 3 is greater than the equilibrium growth rate of the other two species, then it will invade. And also like the previous case, as long as *σ*_33_ < *σ*_23_ or *σ*_33_ < *σ*_13_, then a new equilibrium between species 3 and one of the other species will exist. However, unlike the previous case, the growth rate of the species not at equilibrium will be higher than the equilibrium growth rate for species 3 and the other species (Fig 3C). Therefore, since any equilibrium between any two species can be invaded by the third, stable three-species coexistence occurs. This is the three species case of what we are calling strict pair-wise negative feedback.

**Fig 4:**
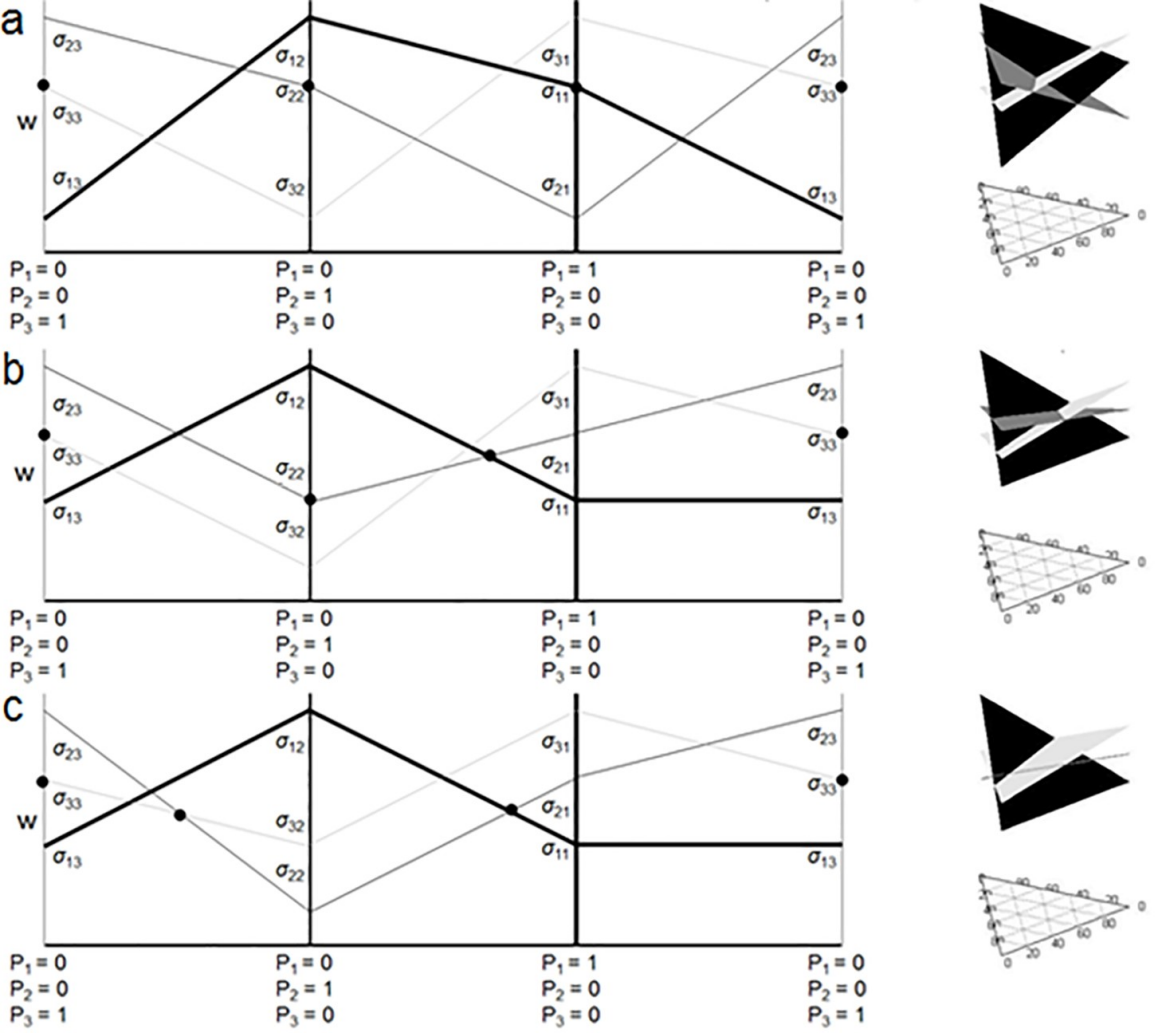
3D triplot of the surface representing the fitness of each species (species 1 = light grey, species 2 = dark grey, species 3 = black) given the relative proportions of each species illustrating how a 3^rd^ species can generate coexistence between 2 species which would otherwise not coexist. a) Shows the case where no species pairs would coexist without the third species, and is completely intransitive. In this example, species 1 replaces species 2, species 3 replaces 1 and species 2 replaces 3. b) Shows the case where species 1 and 2 would coexist in the absence of species 3 due to negative feedbacks, yet even though species 3 would coexist with neither in isolation, all three do coexist through a combination of direct and indirect negative feedbacks. c) Shows the case where the pair-wise combinations of species 1 and 2 and 2 and 3 would coexist, but even though 1 and 3 would not, all three do. Panels a, b, and c are 2D representations of the corresponding 3D plots showing the edge conditions.

Alternatively, the invasion of species 3 could enable species 1 and 2 to coexist in the presence of species 3 when they would not otherwise coexist in the presence of only each other (Fig 4). Thus it is not necessary for species 1 and 2 to coexist in isolation to allow all three to coexist. In the simplest case, for any of the two-species communities, coexistence would not be possible since *σ*_*ii*_ > *σ*_*ij*_ and *σ*_*ij*_ > *σ*_*jj*_ (see Fig 1). Biologically, this inequality indicates that each species effect on its own growth is less than its effect on a competitor’s growth. However, because the third species is always able to invade following the exclusion, 3-species coexistence occurs. This results in the familiar rock-paper-scissors dynamics and we refer to this as strict intransitivity. Kulmatiski et al. (2011) had illustrated this possibility in their Fig 2B, calling it coexistence with positive feedback. We note that it is not actually positive feedback, though pairs of species may have positive feedback in the absence of the third species. Strict pair-wise negative feedback and intransitivity are actually just special cases of negative frequency dependent feedback in the general sense at the community level. Remembering that strict negative feedback is required for coexistence with only 2 species, and that Fig 4 demonstrates that it is not necessary with 3 or more species, this example illustrates how more complex communities can provide more ways for species to coexist through intransitive networks,

The community illustrated in Fig 4A, is an example of only one end of a spectrum of stably coexisting communities. This spectrum ranges from strictly intransitive networks to networks characterized by strict pair-wise negative feedback. In strictly intransitive networks, no pairs of species in the community would coexist without the third species. In contrast, in networks characterized by strict negative pair-wise negative feedback every pair of species would coexist regardless of the third species, because every species does worse in the presence of itself than in the presence of any other species. Fig 4B and 4C show examples of other intermediate community types that allow coexistence, but are stabilized partially by both intransitivity and strict pair-wise negative feedbacks. In each of these cases some pairs of species would coexist in isolation, but not all would. However, the presence of a third species stabilizes the community and allows for three-way coexistence. For example, in panel 4b species 2 and 3 could not coexist in isolation. Species 2 grows better in both its own soil and that of species 3. However, species 1 can invade species 2 and stably coexist with it. Yet, species 3 can invade when species 1 and 2 are stably coexisting, thus, all three are able to coexist. In panel 4c, species 3 would exclude species 1 in isolation. However, species 2, is able to invade and coexist with both 1 and 3 in isolation. Thus, in this case species 2 is acting like a “keystone competitor” since its presence necessary for the stability of 3-way coexistence in the community.

### Stability and mechanisms of coexistence

The spectrum of community coexistence types, that is the number of species with strict pairwise negative feedback within the community, determines the stability of community dynamics. While negative frequency dependent feedbacks can generate oscillations, these oscillations are damped for strict pair-wise negative feedback leading to a stable coexistence equilibrium (Appendix A). In contrast, it has been observed that oscillations of communities structured completely by strict intransitivity are sustained (as identified by Kulmatiski et al. 2011, see also Allesina and Levine 2011). We find that the limited amounts of strict pair-wise negative feedback within the intermediate community coexistence types are sufficient to dampen the oscillations in three-species communities.

The coexistence type of a system also determines the possible impacts of invasion and species extinction. A community that is entirely structured by strict pair-wise negative feedbacks will be completely robust to extinction since the loss of any one species will not change the fact that each species does worst in the presence of itself. Likewise, an invader who uniformly does worse in the presence of itself compared to the presence of the other native species in the community will likely become naturalized and will likely not drive other members of the community extinct. On the other end of the spectrum, a community that is entirely structured by intransitivity will be much more sensitive to extinction since the loss of any one species will result in secondary extinction (see e.g. Allesina and Levine 2011) and thus a complete collapse of diversity in the community. This cascading loss of diversity in the community is inevitable since no two species can coexist without the presence of another species. Communities characterized by intermediate coexistence types will have intermediate levels of robustness to invasion and species loss. In these latter communities certain species can be thought of as “keystone competitors” species since their loss from the system has a much larger impact on community stability than the loss of other species in the system. This can be seen in Fig 4C where the loss of species 2 would result in a monoculture of species 3, but the loss of either species 1 or 3 would result in the stable coexistence of the remaining two species. As communities contain more and more species, the variation in importance of any one species would be expected to increase. Notice that the species remaining after an extinction event in a community structured by partially intransitive networks are the species coexisting by strict pair-wise negative feedbacks.

This analysis of three-species communities shows that with increasing species richness community dynamics no longer depend solely on the pair-wise interaction coefficients. Instead, the details of competition with third parties can be critical to overall dynamics through the creation of intransitive networks. As more species are added beyond three, these multi-species pathways may become more important, which will be examined in the next section using simulations of theoretical and experimental plant communities.

### Coexistence in theoretical and real experimental communities

**Fig 5:**
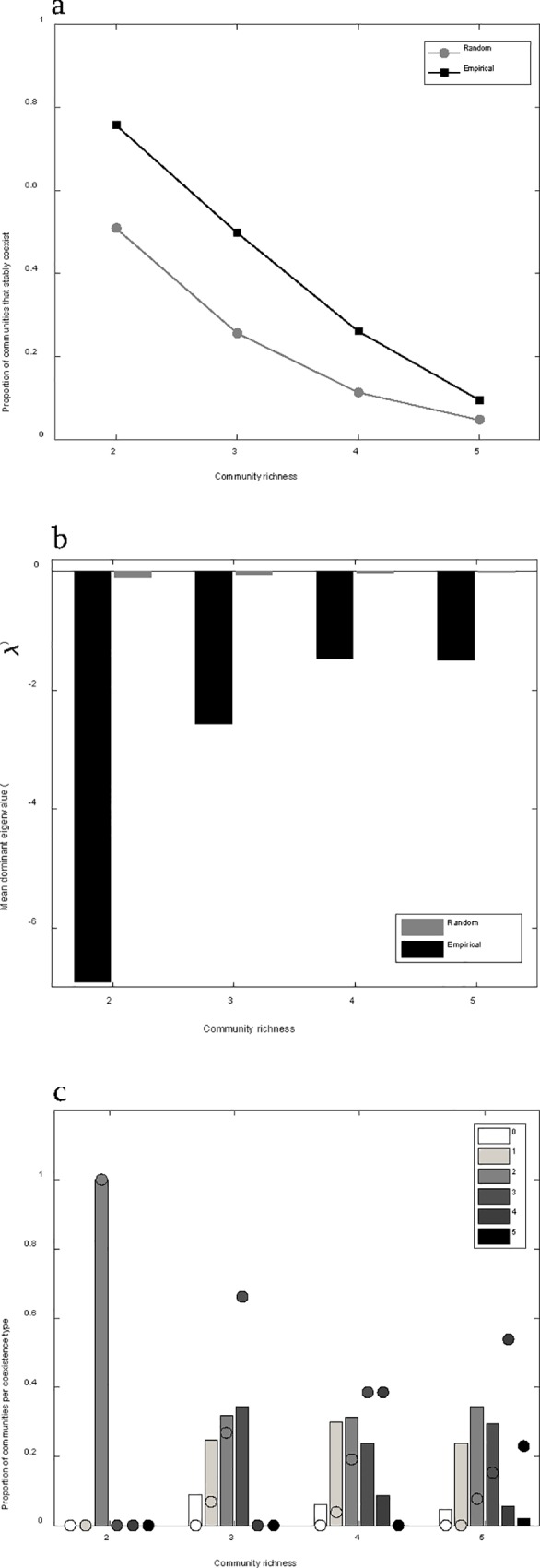
Comparison of randomly assembled communities and communities assembled from the literature reporting plant-soil feedback experiments. a) A comparison of the proportion of assembled communities that had a feasible and stable coexistence equilibrium for random (grey line) and experimental (black line) data. Communities assembled at random were less likely to contain a feasible and stable equilibrium point as compared to communities assembled from published experimental plant-soil feedback data. Although coexistence becomes less likely as species richness increases, communities assembled from published feedback data remained more likely to fully coexist than communities assembled at random. b) A comparison of the mean dominant eigenvalues of feasible community coexistence equilibrium points between communities assembled from published data (black bars), and assembled from random data (grey bars). c) Overlay of the histograms of the distribution of community coexistence types by species richness for randomly assembled communities (bars) and communities assembled from published data (circles). As community richness increases, so do the number of qualitatively different community structures that permit coexistence. The shading represents the range of coexistence types with 0 (white) corresponding to strict intransitivity and each number above 0 represents the number of species with strict pair-wise negative feedback. With increasing community richness, communities assembled from published data disproportionately coexist through strict pair-wise negative feedback.

We found that coexistence was much more common in the literature data than would be expected from randomly assembled communities (Fig 5A, $\chi_{1}^{2}$ = = 38.4, 67.2, 37.6, 12.2, for 2, 3, 4, and 5 species sub-samples respectively, p < 0.0001 for 2, 3 and 4 species, p = 0.0002 for 5 species). Moreover, real parts of the dominant eigenvalue were much lower for experimental coexisting communities than randomly assembled communities, showing that the former were more resilient (Fig 5B). We also found that coexistence in communities assembled from published data was maintained more often by strict pair-wise negative feedbacks than would be expected based on randomly assembled communities of 3, 4 and 5 species (Fig 5A, $\chi_{1}^{2}$ = 42.1, 39.5 and 12.9, for 3, 4, and 5 species sub-samples respectively, p < 0.0001 for 3 and 4 species, p = 0.0002 for 5 species). Although strict pair-wise negative feedback is the only stable coexistence type for 2-species communities, and most frequently observed coexistence type in 3-species communities (for both random and experimental data), the proportion of communities persisting through alternative coexistence types increased for communities consisting of more species (with the exception of strict intransitivity, Fig 5C). This observation emphasizes the potential importance of keystone competitors for maintaining coexistence in real communities.

### Resistance to invasion and robustness to species loss

**Fig 6:**
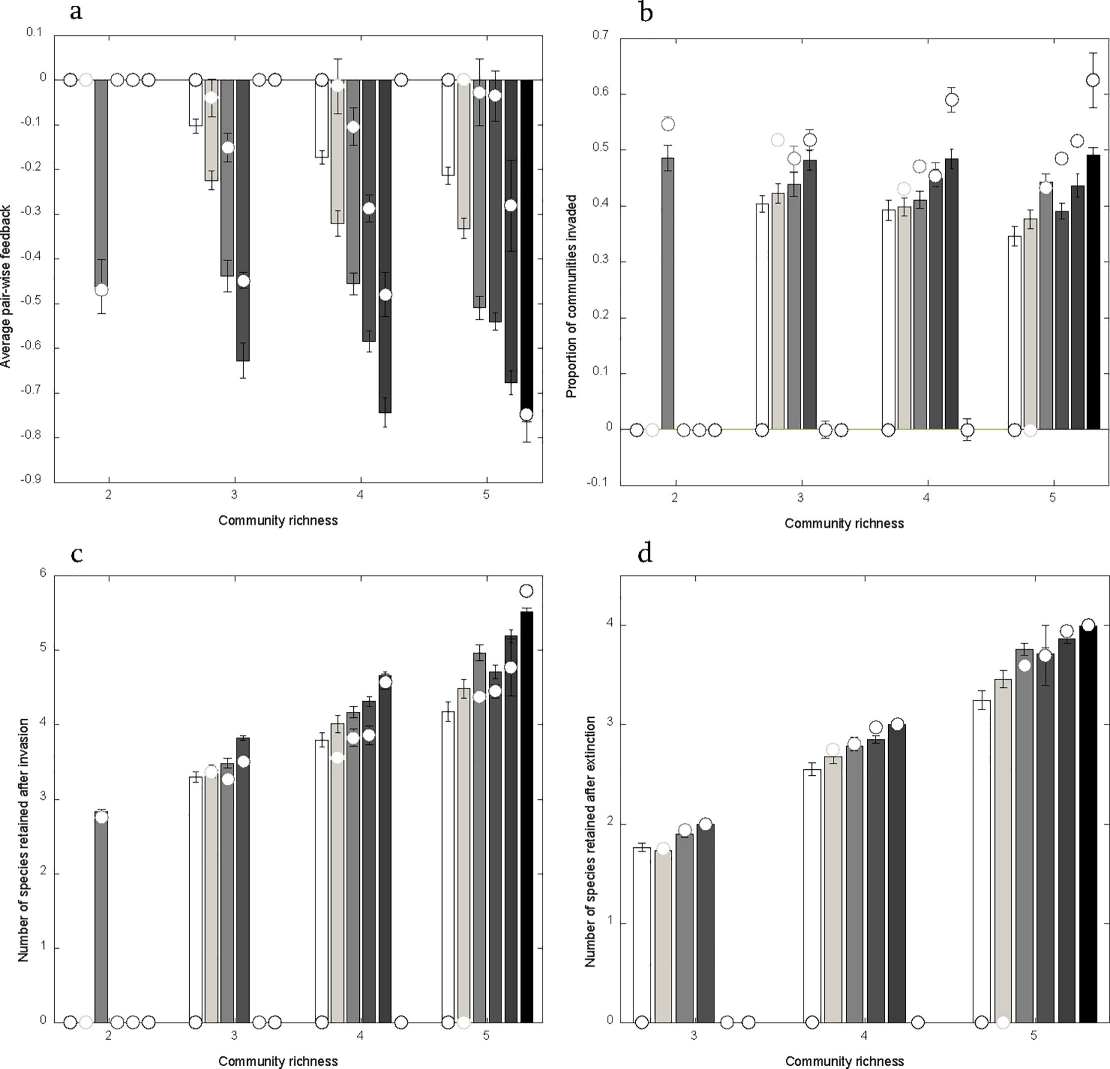
Community resistance to invasion and robustness as a function of coexistence type. All panels show comparisons between randomly assembled communities (bars) and communities assembled from published data (circles). The shading represents the range of coexistence types with 0 (white) corresponding to strict intransitivity and each number above 0 represents the number of species with strict pair-wise negative feedback. a) Average pair-wise feedback was increasingly negative with community richness, and were most strongly negative for randomly assembled communities. b) Communities structured by strict pair-wise negative feedback could be most easily invaded by new species. c) Communities structured by strict pair-wise negative feedback could retain most species after a successful invasion event. d) Communities structured by strict pair-wise negative feedback could retain most species after an extinction event.

Average feedback became more negative as richness of randomly assembled communities increased, and average feedback was often less negative for experimental communities than for randomly assembled communities (Fig 6). This result suggests that indirect interactions in experimental communities have a stabilizing effect at the community level (Fig 5C, Fig 6A). Communities structured by strict intransitive feedback were least susceptible to invasion by new species, whereas communities structured by strict pair-wise negative feedback were most susceptible to invasion (Fig 6B). Furthermore, experimental communities were slightly more susceptible to invasion than randomly assembled communities (Fig 6B). Invasions had the most negative effects on resident species in communities structured by strict intransitivity, and the least negative effects on resident species in communities structured by strict pair-wise negative feedback (Fig 6C). Finally, we found that secondary extinctions (after removal of a resident species) occurred most often in communities structured by strict intransitivity, whereas secondary extinctions did not occur in communities structured by strict negative pair-wise feedback (Fig 6D).

## Discussion

Previous analytical models show that frequency dependent feedbacks can facilitate plant coexistence in simple two-species communities [11,12,18]. The general framework presented here extends the conditions for plant coexistence mediated by frequency dependent feedbacks beyond two species. Kulmatiski et al. (2011) used a three-species plant-soil feedback model to illustrate coexistence without strict pair-wise negative feedback, but a comprehensive analysis was beyond the scope of that study. Eppinga et al. (2018) present an analytical solution to the conditions for coexistence via plant soil feedbacks in multispecies communities that identified the possibility of intransitive networks. Our theoretical analyses of multispecies communities more completely describe qualitatively new community structures that can generate species coexistence. Through intransitivity, multispecies communities can coexist when individual pairs of species would not otherwise. We found that communities structured by strict pair-wise negative feedbacks and those structured by strict intransitivity are the end points of a continuous spectrum of community coexistence types. As the richness of communities increases, more intermediate community types, which are stabilized by a combination of strict pair-wise negative feedbacks and intransitivity, become theoretically possible. We found that where a community lies on this continuum has important consequences for its stability to perturbation (Fig 6). For instance, communities completely structured by pair-wise negative feedbacks will be extremely robust to extinction since the loss of any given species will not affect the coexistence of the other species in the community (Fig 6). On the other hand, a community completely structured by intransitivity will be much more susceptible to extinction since the loss of any given species may result in a cascading extinction of competitors. We found that communities randomly assembled with greater richness were more likely to be at least partially structured through intransitivity (Fig 5), and therefore would be more vulnerable to cascading extinction events, which is consistent with previous results using a Lotka-Volterra model [22]. As richness increases, the average dominant eigenvalue becomes less negative, meaning that strict pair-wise negative feedback becomes less and less common as the number of species in randomly assembled community increases. This could explain why we found that communities assembled from previously published feedback studies were more likely than randomly assembled communities to coexist through strict pair-wise negative feedbacks rather than intransitivity (Fig 5). Interestingly, we found that the enhanced stability of observed communities was partly due to indirect interactions, rather than more negative average pair-wise feedbacks (Fig 6A). Non-random patterns of interaction strengths, contributing to network stability has previously been identified in real food webs [52,53]. This study suggests that plant-soil feedbacks provide a similar mechanism for stability in competitive networks.

Our graphical analysis of three species models showed that a consequence of coexistence through frequency dependent partial intransitivity is the existence of intra-guild keystone species (Figs 2 and 3). Although analytical description of this phenomenon for multi-species is challenging, numerical simulations suggested that the same principle applies here. Specifically, we observed more disruptive effects of species introduction and removal in multi-species communities with (partial) intransitivity, suggesting the presence of species with disproportionate effects on community stability (Fig 6). Just as food webs can be stabilized by keystone predators [54] it is possible that plant communities can be stabilized by keystone competitors. As noted above, the presence of such keystone species will generate variability among communities in their stability and therefore robustness to species loss. Since there are more combinations of pair-wise growth parameter values that lead to naturalization in communities structured by intransitivity than those structured by strict pair-wise negative feedbacks, the species being added through speciation or immigration are more likely to coexist through intransitivity. However, a community stabilized by a keystone competitor is likely to leave only the portion of the community structured by strict pair-wise negative feedbacks following an extinction event (Fig 4). This generates a tension between the increased likelihood of adding species maintained through intransitivity and the increased likelihood that species maintained by intransitivity will subsequently be lost following invasion and extinction events. Over time, as communities gain and lose species through succession, the ones coexisting through strict pairwise negative feedbacks will be more likely retained. Therefore, we expect that later successional communities would be more likely structured through strict pair-wise negative feedbacks than early successional communities. Given that species coexisting through strict pair-wise negative feedbacks always perform the worst in the presence of themselves relative to heterospecifics, when their net feedback across the community is averaged they tend to have more negative average feedbacks than species coexisting through intransitivity. This correlation between community structure driven feedback magnitudes and community age could contribute to the understanding of recent empirical work which has shown that dominant invaders have less negative average feedbacks than natives [9,55,56], as well as predicts a general pattern between successional stage and average feedback. However, further studies would be required to attribute the pattern to community coexistence type.

Our results contribute to a growing body of evidence that plant communities are structured by frequency dependent negative feedbacks, and that plant-soil interactions may create these feedbacks [8,9,14]. We found that natural communities studied in plant-soil feedback experiments are more likely to demonstrate coexistence (Fig 5), and are particularly more likely to coexist through strict pair-wise negative feedbacks than expected by chance (Fig 5). In fact, published data suggest that strict intransitivity and the associated unstable population oscillations are very unlikely, and that natural communities are more resilient to invasion and extinction than randomly assembled communities (Fig 6). Our finding that real PSFs are more likely to have strong pair-wise negative feedbacks than expected by chance is consistent with strong trade-offs governing the dynamics of virulence and mutualism and the degree of specialization of plant-microbe interactions [57,58]. The robustness of this analysis is limited by the small number of full-factorial feedback studies, and highlights the need for greater number of empirical studies that use a full-factorial test experiment.

While natural communities are significantly different than those assembled at random, the tendencies of randomly assembled communities may inform our understanding of nature. For example, we found that as community richness increases, while there are more qualitatively different routes to coexistence, stably coexisting communities are more and more difficult to generate by chance (Fig 5). This result is consistent with the results of previous work based on a Lotka-Volterra modeling approach [22]. As a consequence, average feedback became more negative overall with increasing species richness (Fig 6). Interestingly, Johnson et al. (2012) found evidence of this relationship, as richness of tree species increased within forests of eastern North America, the average feedback of species within these forests became more negative. Our results provide theoretical demonstration of how communities assembled with feedbacks could generate such a pattern.

This model provides a general framework for understanding plant community dynamics and a mechanism for many of the hypotheses used to explain invasion, succession and coexistence. Available data suggest that negative feedbacks are more common in nature than expected by chance, and as a consequence natural communities will be more stable, both in numerical dynamics and in resilience to extinction. However, our analysis highlights the need for more full factorial tests of PSFs [e.g. 8,11,15,25,34,44] in order to confirm the generality of this conclusion. More generally, our results demonstrate how measurements of interactions mediated through the soil community can be extended to gain inference on invasion dynamics and community stability in plant communities.

## Appendix A

### Analytical analyses and numerical simulations of multi-species communities

The species densities at the internal coexistence equilibrium (Eq 10 of the main text) can be substituted in the Jacobian matrix of the system:

$$J=\left[\begin{array}{cccc} \frac{\partial F_{1}}{\partial P_{1}} & \frac{\partial F_{1}}{\partial P_{2}} & .. & \frac{\partial F_{1}}{\partial P_{n}}\\ \frac{\partial F_{2}}{\partial P_{1}} & .. & .. & ..\\ .. & .. & .. & ..\\ \frac{\partial F_{n}}{\partial P_{1}} & .. & .. & \frac{\partial F_{n}}{\partial P_{n}} \end{array}\right]$$ (A1)

With: $F_{i}=P_{i}\left(w_{i}-\sum_{j=1}^{n}w_{j}P_{j}\right)$. The leading eigenvalue of this Jacobian matrix (i.e. the eigenvalue that has the maximal real part) evaluated at the coexistence equilibrium point determines its local stability. Within the range of *σ* coefficients considered (between 0 and 1, meaning that negative to neutral effects are considered), the coexistence equilibrium can be attracting, neutrally stable (requiring an odd number of species and parameter symmetry) or repelling (S1 Fig).

In cases where the internal coexistence equilibrium is repelling, the leading eigenvalue of the Jacobian matrix is positive. Under these conditions, only a subset of the plant species community may survive, but there is also a possibility that none of the plant species get excluded, In this latter case, all species remain present, but alternate in frequency via a heteroclinic cycle (S1 Fig). To what extent persistence of all species through heteroclinic cycles can occur in real communities is an interesting point of discussion. As individual species may repeatedly be driven to very low densities during a heteroclinic cycle (S1 Fig), local extinction may become likely in real communities (Revilla et al. 2013). On the other hand, one could argue that within a spatial context, this risk of extinction may be counteracted by the repeated re-immigration of locally extinct species (Revilla et al. 2013).

This comparison between analytical calculations of the system’s leading eigenvalue and numerical simulations shows that the magnitude of the dominant eigenvalue can indicate the degree of stability and resilience of the coexistence equilibrium [e.g. 39], but species persistence needs to be examined via numerical integration for cases in which the real part of the dominant eigenvalue is positive. Hence, these approaches were combined in the analyses comparing randomized and empirical communities in the main text.

## Supporting information

S1 Fig Illustrations of the possible properties of the internal coexistence equilibrium of the frequency dependent feedback model (Eq 6 in the main text) with three species (species 1: dotted line; species 2: dash-dotted line; species 3: full line). In all four cases, the internal coexistence equilibrium is feasible (equations 13a and 13b in the main text). a) The coexistence equilibrium is an attractor (M = [0.2 0.85 0.2; 0.2 0.05 0.5; 0.8 0.25 0.1], dominant eigenvalue, λ = -0.10 + 0.13i). b) The coexistence equilibrium is neutrally stable (M = [0.5 0.85 0.2; 0.15 0.5 0.75; 0.8 0.25 0.5], λ = 0 + 0.17i). c) The coexistence equilibrium is unstable, and the system develops to an equilibrium containing a subset of the plant species pool (M = [0.9 0.85 0.25; 0.15 0.9 0.8; 0.75 0.2 0.9], λ = 0.13 + 0.17i). d) The coexistence equilibrium is unstable, but all species persist, by means of a heteroclinic cycle (M = [0.5 0.85 0.2; 0.25 0.5 0.4; 0.8 0.2 0.35], λ = 0.01 + 0.10i).
(TIF)
