# Supplementary figures and images for: Plant-soil feedbacks promote coexistence and resilience in multi-species communities
Source: PLoS One. 2019 Feb 11;14(2):e0211572. doi: 10.1371/journal.pone.0211572 (PMC6370276; doi:10.1371/journal.pone.0211572)

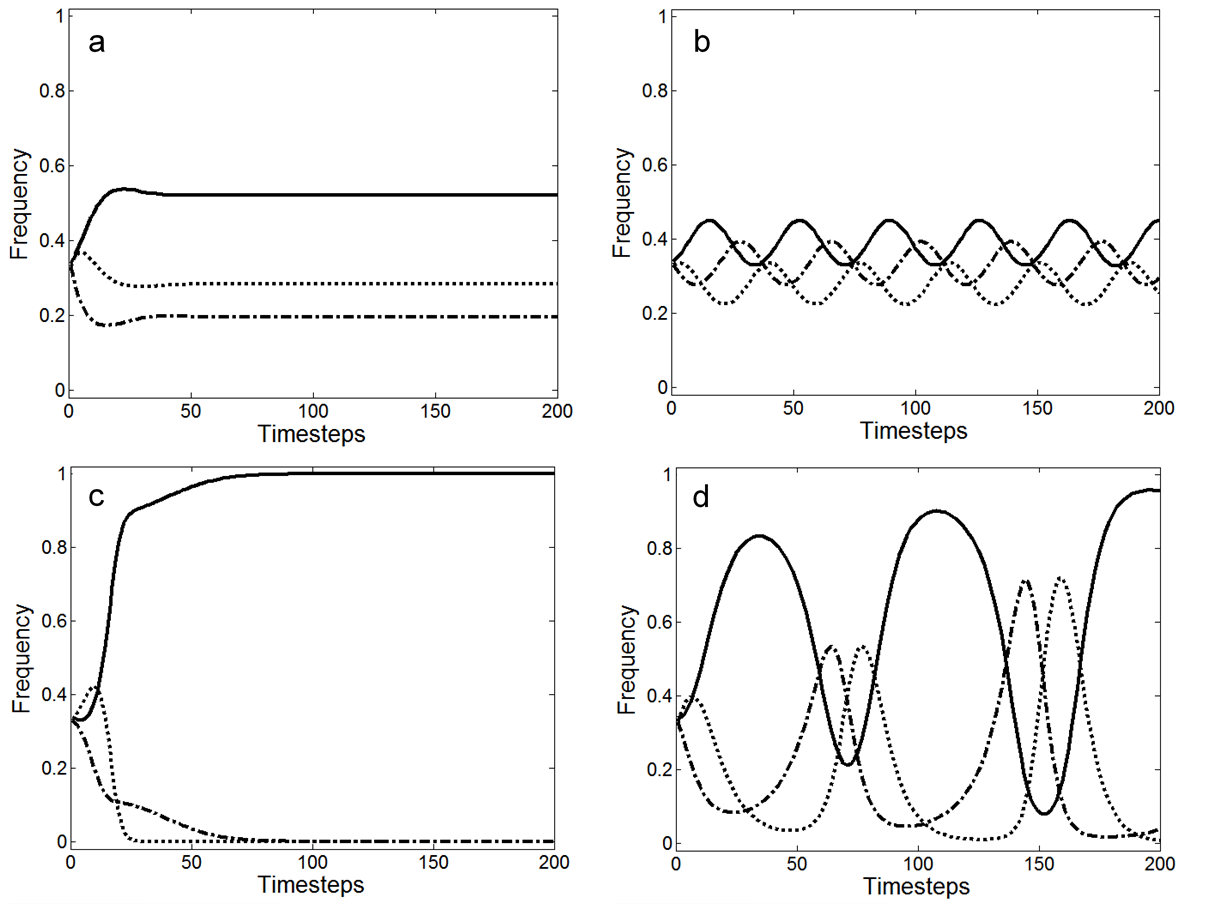

Supplement: S1 Fig — In all four cases, the internal coexistence equilibrium is feasible (equations 13a and 13b in the main text). a) The coexistence equilibrium is an attractor (M = [0.2 0.85 0.2; 0.2 0.05 0.5; 0.8 0.25 0.1], dominant eigenvalue, λ = -0.10 + 0.13i). b) The coexistence equilibrium is neutrally stable (M = [0.5 0.85 0.2; 0.15 0.5 0.75; 0.8 0.25 0.5], λ = 0 + 0.17i). c) The coexistence equilibrium is unstable, and the system develops to an equilibrium containing a subset of the plant species pool (M = [0.9 0.85 0.25; 0.15 0.9 0.8; 0.75 0.2 0.9], λ = 0.13 + 0.17i). d) The coexistence equilibrium is unstable, but all species persist, by means of a heteroclinic cycle (M = [0.5 0.85 0.2; 0.25 0.5 0.4; 0.8 0.2 0.35], λ = 0.01 + 0.10i). (TIF) [file pone.0211572.s001.tif]
